# Analysis of lumbar vertebrae fractures among inpatients in a primary hospital: A 10-year epidemiological study

**DOI:** 10.1097/MD.0000000000030111

**Published:** 2022-08-19

**Authors:** Dingding Jia, Xin Qiao, Dongwei Wu, Zhanfeng Song, Jianqing Ma, Ke Yang, Xiufang Mo, Zhanyong Wu

**Affiliations:** a Orthopedics Department, Xingtai Orthopaedic Hospital, Xingtai City, Hebei Province, China.

**Keywords:** age, epidemiological characteristics, fracture mechanism, fracture time, gender, lumbar fracture

## Abstract

**Background:**

To analyze the epidemiological characteristics and changing trends of lumbar fractures in Xingtai Orthopedic Hospital in the past 10 years, and to improve the prevention and treatment of lumbar fractures.

**Methods:**

Using the hospital information system, data on patients with lumbar fractures in our hospital from 2009 to 2018 were collected regarding their age, gender, fracture time, injury mechanism, and the type of fracture. The epidemiological characteristics and trends of lumbar fractures for the period were summarized and analyzed.

**Results:**

The age of male patients with a high incidence of lumbar fractures was 61 to 70 years, followed by 51 to 60 years. The age of female patients with the highest incidence rate was 61 to 70 years, followed by 51 to 60 years (19.22%). Lumbar fractures in group A were predominantly of men. The majority of lumbar fractures in group B were of women. In group A, the incidence rate was higher in young men (21–50 years) than in women and higher in women >51 years. Most of the affected individuals were women. In group B, there were more middle-aged and young men (21–50 years) than women; however, there were more women than men aged ≥51 years. Car accident injury was the main cause of fractures, but in group B women, low-energy injuries were the main cause of fractures. The periods of high incidence in groups A and B were 4 to 6 years and 7 to 9 years, respectively. The number of injuries in group A was the highest and burst fracture was the main fracture type. In group B, the number of fall injuries was the highest, followed by car accident injuries, and compression fracture was the main fracture type.

**Conclusion:**

The number of lumbar fractures in women caused by low-energy injuries showed an increasing trend. The type of compression fracture increased, which might be related to osteoporosis caused by the decrease in the estrogen level after menopause.

## 1. Introduction

In a review, it was shown that the incidence of spinal fractures varies widely around the world, ranging from 10.4 PMI (per million inhabitants) in the Netherlands to 83 PMI in Alaska; however, the incidence is typically around 15 to 30 PMI.^[1]^ A separate assessment in 2014 showed that the global PMI was 23 in 2007.^[2]^ Lumbar fracture is the most common clinical spinal fracture, with many pathogenic factors, complex injury, and a high disability rate. It causes serious psychological and economic stress to patients and their families.^[3]^ Leucht et al^[4]^ in the United States, found in a study with 562 people with spinal fractures that lumbar fractures accounted for 50.4% of all spinal fractures. Many studies have been conducted on the epidemiology and surgical methods of postoperative rehabilitation of lumbar fractures, but there are few studies on the age, gender, injury time, injury mechanism, and the classification of fractures of patients with lumbar fractures. In this study, 4855 medical records were collected in our hospital for 10 years, and their changing trends were summarized and analyzed. We investigated risk factors that might help to develop preventive measures in the future.

An important difference between this study and most other epidemiological studies is that the First Xingtai Orthopedic Hospital is a prefecture-level city hospital in Hebei Province, China, and is an important representative of grassroots hospitals in China. The local population density is relatively high, and the city is an agglomeration of industry, transportation, and agriculture. The second difference is that this study included basic information of patients with lumbar fractures in the past 10 years, that is, the data analyzed was collected over a long time. Through comparative analysis, the change in the incidence trend of lumbar fractures in local primary hospitals could be determined.

## 2. Data and Methods

### 2.1. Clinical data

Xingtai City is a medium-sized city in Hebei Province, China. Xingtai Orthopedic Hospital is a local orthopedic specialist hospital and receives the highest number of patients with lumbar fractures every year. The clinical data of 4855 patients with lumbar fracture admitted to our hospital (including nonsurgical treatment and surgical treatment) from 2009 to 2018 were retrospectively analyzed. The inclusion criteria were: patients who were ≥16 years, and they were diagnosed with lumbar fracture by imaging data. The exclusion criteria were: patients <16 years; individuals with old fractures and countercheck; the diagnosis was not clear in the imaging data; the medical records were incomplete.

### 2.2. Research methods

We retrieved inpatient information through the hospital information system and collected data on the age, gender, fracture time, injury mechanism, and fracture type of 4855 patients admitted to our hospital from 2009 to 2018 with lumbar fractures. The information of the patients from 2009 to 2013 was placed in group A, and the information of the patients from 2014 to 2018 was placed in group B. The data of the 2 groups of patients were compared. The indices included in the analysis were age, gender, fracture time, and fracture mechanism, which included traffic accident injury, high-energy fall injury (*h* > 2 m), high fall injury (*h* < 2 m), stumbling injury, heavy object injury, and sprain. Fracture classification was performed by Denis classification, which categorizes fractures into 4 types. Type I is a compression fracture, type II is a burst fracture, type III is a distractive flexion injury, and type IV is a fracture with dislocation. The patients were divided into 8 age classes, which included 16 to 20 years, 21 to 30 years, 31 to 40 years, 41 to 50 years, 51 to 60 years, 61 to 70 years, 71 to 80 years, and >80 years. This study was approved by the Institutional Review Board of Xingtai Orthopedic Hospital.

### 2.3. Statistical analysis

The SPSS 19.0 (Statistical Product and Service Solutions 19.0, Chicago, IL) statistical software was used to represent continuous data as means and standard deviation. Count data of patients in the 2 groups, such as gender, different fracture mechanism, time period, injury mechanism, and fracture types, were compared by the *χ*^2^ (chi-square) test. The differences between the 2 groups were considered to be statistically significant at *P* < .05.

## 3. Results

### 3.1. Overview of patient data

A total of 4855 patients were included in this study (see Table 1 for details), with 2383 males (49.08%) and 2472 females (50.92%); the male-to-female ratio was 0.96:1. Their age ranged from 16 to 95 years, with an average age of 55.5 years. Men who were 61 to 70 years old (18.76%) had a high incidence of lumbar fractures, followed by those who were 51 to 60 years old (17.71%). For women, the most frequent age group with lumbar fractures was 61 to 70 years (21.32%), followed by 51 to 60 years (19.22%).

**Table 1:** The age distribution of the patients (n = 4855) with lumbar fractures.

Age bracket (yr)	Man	Woman	Total
16–20	142 (5.96%)	86 (3.48%)	228 (4.70%)
21–30	239 (10.03%)	198 (8.01%)	437 (9.00%)
31–40	285 (11.96%)	208 (8.41%)	493 (10.15%)
41–50	303 (12.72%)	254 (10.28%)	557 (11.47%)
51–60	422 (17.71%)	434 (17.56%)	856 (17.63%)
61–70	447 (18.76%)	527 (21.32%)	974 (20.06%)
71–80	332 (13.93%)	406 (16.42%)	738 (15.20%)
≥81	213 (8.94%)	359 (14.52%)	572 (11.78%)
Total	2383	2472	4855

### 3.2. Age and sex distribution of the patients in the 2 groups

There were 766 males and 624 females in group A, and the ratio of males to females was 1.23:1. There were 1617 males and 1848 females in group B, with a male-to-female ratio of 0.88:1. Lumbar fractures in group A were predominantly of males. Lumbar fractures in group B were more common in women. In group A (21–50 years), the cumulative fracture composition ratio was 37.21% in males and 33.82% in females, which was higher in males than in females. The cumulative fracture ratio for women >51 years was 60.41%, while that for men was 57.19%, and the ratio was higher in women than in men. Most of the fractures occurred in individuals who were 61 to 70 years (250/1390, 17.99%), and mostly occurred in women (117/624, 18.75%). In group B, the cumulative proportion of young and middle-aged (21–50 years) men was 33.52%, while that of women was 24.29%, and the number of male patients was more than the number of female patients. The cumulative fracture ratio of individuals >61 years was 73.00% in women and 60.37% in men. Most of the patients aged 61 to 70 years were female (410/1848, 22.19%). The male and female composition ratio of group A was significantly lower than that of group B (*χ*² = 6.882, *P* < .05, *χ*² = 7.231, *P* < .05, *χ*² = 10.239, *P* < .05, *χ*² = 14.430, *P* < .01) among individuals who were 51 to 60 years old, 61 to 70 years old, 71 to 80 years old, and ≥81 years old (see Table 2 for details).

**Table 2:** The age distribution of patients with lumbar fractures in groups A and B.

Age bracket (yr)	A			B			*χ* ^2^	*P*
	Man	Woman	Total	Man	Woman	Total		
16–20	43 (5.61%)	36 (5.77%)	79 (5.68%)	99 (6.12%)	50 (2.71%)	149 (4.30%)	3.171	.075
21–30	93 (12.14%)	62 (9.94%)	155 (11.15%)	146 (9.03%)	136 (7.36%)	282 (8.14%)	2.732	.098
31–40	102 (13.32%)	78 (12.50%)	180 (12.95%)	183 (11.32%)	130 (7.03%)	313 (9.03%)	0.152	.697
41–50	90 (11.75%)	71 (11.38%)	161 (11.58%)	213 (13.17%)	183 (9.90%)	396 (11.43%)	0.206	.650
51–60	119 (15.54%)	89 (14.26%)	208 (14.96%)	303 (18.74%)	345 (18.67%)	648 (18.70%)	6.882	<.005
61–70	133 (17.36%)	11 (18.75%)	250 (17.99%)	314 (19.42%)	410 (22.19%)	724 (20.89%)	7.231	<.005
71–80	101 (13.19%)	82 (13.14%	183 (13.17%)	231 (14.29%)	342 (17.53%)	555 (16.02%)	10.239	<.005
≥81	85 (11.10%)	89 (14.26%)	174 (12.52%)	128 (7.92%)	270 (14.61%)	398 (11.49%)	14.43	<.005
Total	766 (100%)	624 (100%)	1390 (100%)	1617 (100%)	1848 (100%)	3465 (100%)		

### 3.3. Distribution characteristics of fracture injury factors and gender of patients in the 2 groups

According to the distribution of fracture mechanisms in the 2 groups of patients, the 3 most frequently occurring injury factors in male group A were traffic accident, high-energy falls and high fall injury, accounting for 25.20%, 22.10%, and 15.54%, respectively (Table 3). For women, traffic accident injury, high fall injury, and stumbling injury were 20.35%, 20.03%, and 16.83%, respectively. In group B, the 3 most frequent injury mechanisms in men were car accident injury, high fall injury, and stumbling injury, which were 25.23%, 22.45%, and 22.76%, respectively. Falling injury, fall injury, and traffic accident were 27.49%, 24.68%, and 22.78%, respectively, in women. According to the overall distribution characteristics, car accident injury was the main cause of injury, but in the incidence of injury in women in group B, a low-energy injury was the main cause of injury, and the male-to-female ratio with fall injury in group B was significantly higher than that in group A (*χ*² = 4.125, *P* < .05).

**Table 3:** The distribution of injury mechanism of lumbar fracture patients in groups A and B.

Injury mechanism	A			B			*χ* ^2^	*P*
	Man	Woman	Total	Man	Woman	Total		
High-energy falls	154 (20.10%)	104 (16.67%)	258 (18.56%)	258 (15.96%)	238 (12.88%)	496 (14.31%)	4.032	<0.05
Traffic accident	193 (25.20%)	127 (20.35%	320 (23.02%)	408 (25.23%)	421 (22.78%)	829 (23.92%)	11.396	<0.001
High falls	119 (15.54%)	12 (20.03%)	244 (17.55%)	363 (22.45%)	456 (24.68%)	819 (23.64%)	1.501	0.221
Heavy object injury	95 (12.40%)	80 (12.82%)	175 (12.59%)	100 (6.18%)	87 (4.71%)	187 (5.40%)	0.024	0.877
Stumbling injury	104 (13.58%)	105 (16.83%)	209 (15.04%)	268 (22.76%)	508 (27.49%)	876 (25.28%)	4.125	<0.05
Sprain	101 (13.19%)	83 (13.30%)	184 (13.24%)	120 (7.42%)	138 (7.47%	258 (7.45%)	3.017	0.082
Total	766 (100%)	624 (100%)	1390 (100%)	1617 (100%)	1848 (100%)	3465 (100%)		

### 3.4. Distribution characteristics of fracture time and sex in the 2 groups

The high incidence of injury in groups A and B was from April to June and from July to September, respectively (Table 4). From April to June, the composition ratio of group B was greater than that of group A (*χ*² = 6.361, *P* < .05), the difference in the sex ratio was statistically significant. From July to September, the composition ratio of group B was greater than that of group A (*χ*² = 12.263, *P* < .001), the difference in the sex ratio was statistically significant. From January to March, the composition ratio of group A was greater than that of group B (*χ*² = 2.867, *P* > .05), and there was no significant difference in the distribution of the sex ratio. From October to December, the composition ratio of group B was greater than that of group A (*χ*² = 3.791, *P* > .05), and the difference in the distribution of the sex ratio was not statistically significant.

**Table 4:** The distribution of the time of injury of lumbar fracture patients in groups A and B.

Time period (mo)	A			B			*χ* ^2^	*P*
	Man	Woman	Total	Man	Woman	Total		
1–3	176 (22.98%)	148 (23.72%)	324 (23.31%)	2 (214.29%)	251 (13.73%)	485 (14.00%)	2.867	.9
4–6	224 (29.24%)	198 (31.73%)	422 (30.36%)	50 (30.85%)	596 (32.60%)	1101 (31.77%)	6.361	<.005
7–9	238 (31.07%)	176 (28.21%)	414 (29.78%)	51 (31.40%)	571 (31.24%)	1085 (31.31%)	12.263	<.001
10–12	128 (16.71%)	102 (16.35%)	230 (16.55%)	38 (23.46%)	410 (22.43%)	794 (22.91%)	3.791	.052
Total	766 (100%)	624 (100%)	1390 (100%)	1637 (100%)	1828 (100%)	3465 (100%)		

### 3.5. Distribution characteristics of Denis typing in injury mechanism in the 2 groups of patients

There was a significant difference in the distribution of Denis fracture types between the 2 groups (*χ*² = 16.417, *P* < .001; Table 5). The number of accident injuries in group A was the highest, and burst fracture was the main fracture type. The second most common injury method was high fall injury, where burst fracture was the main fracture type. Fall injury was the most common injury method in group B, with compression fracture being the main fracture type, followed by car accident injury. There was a significant difference in the distribution of fracture types between groups A and B in traffic accident injuries (*χ*² = 13.186, *P* < .05). In high fall injuries, the distribution of fracture types between the 2 groups was similar (*χ*² = 3.184, *P* > .05). Additionally, in the case of low-energy injuries, there was a significant difference in the distribution of fracture types between groups A and B in the case of stumbling injury (*χ*² = 12.538, *P* < .05) and in the case of high fall injury (*χ*² = 13.172, *P* < .05; see Table 5 for details).

**Table 5:** The distribution of the fracture type composition ratio in groups A and B.

Injury mechanism	A					B					*χ* ^2^	*P*
	Compression fracture	Burst fracture	Flexion-distraction fracture	Fracture dislocation	Total	Compression fracture	Burst fracture	Flexion-distraction fracture	Fracture dislocation	Total		
High-energy falls	68 (9.38%)	75 (22.52%)	52 (37.41%)	63 (32.64%)	258 (18.56%)	133 (6.81%)	172 (19.95%)	87 (31.75%)	104 (27.66%)	496 (14.31%)	3.184	.364
Traffic accident	80 (11.03%)	104 (31.23%	71 (51.08%)	65 (33.68%)	320 (23.02%)	295 (15.10%)	241 (27.96%)	137 (50.00%)	156 (41.49%)	829 (23.92%)	13.186	<.05
High falls	138 (19.03%)	72 (21.62%)	6 (4.32%)	28 (14.51%)	244 (17.55%)	548 (28.06%)	172 (19.95%)	34 (12.41%)	65 (17.29%)	819 (23.64%)	13.172	<.05
Heavy object injury	77 (10.62%)	54 (16.22%)	10 (7.19%)	34 (17.62%)	175 (12.59%)	82 (4.20%)	63 (7.31%)	14 (5.11%)	28 (7.45%)	187 (5.40%)	1.701	.637
Stumbling injury	178 (24.55%)	28 (8.41%)	0 (0.00%)	3 (1.55%)	209 (15.04%)	645 (33.03%)	207 (24.019%)	2 (0.73%)	22 (5.85%)	876 (25.28%)	12.538	<.005
Sprain	184 (25.38%)	0 (0.00%)	0 (0.00%)	0 (0.00%)	184 (13.24%)	250 (12.80%)	7 (0.81%)	0 (0.00%)	1 (0.27%)	258 (7.45%)	5.931	<.005
Total	725 (100%)	333 (100%)	139 (100%)	193 (100%)	1390 (100%)	1953 (100%)	862 (100%)	274 (100%)	376 (100%)	3465 (100%)		

## 4. Discussion

Spinal fractures seriously affect the quality of life of the patients and also cause great psychological and economic stress among individuals, families, and society. The incidence of spinal fracture accounts for 5% to 6% of total body fractures, and lumbar spine fractures are the most prevalent of all spinal fractures.^[5–9]^ Due to its physical structure, the lumbar spine does not get thoracic support. When subjected to external force, the lumbar spine independently forces and the high-energy external force cannot be buffered, causing fractures during high-energy injuries. Older people are also prone to fractures through low-energy injuries due to osteoporosis and other causes. Lumbar fracture is common clinically. Studies have found that the incidence of lumbar fracture is about 7.39/100,000 years in young people (<60 years old) and 56.78/100,000 years in old people.^[9]^ Lumbar fracture often causes neurological symptoms, and because of its location in the body, it significantly affects the patient’s life after injury. This study collected data on adult lumbar fractures in our hospital during the past 10 years, to provide information for the prevention and treatment of lumbar fractures.

The results of the analysis of the distribution of age and gender showed that the number of patients with lumbar fractures increased significantly from 2009 to 2013 and from 2014 to 2018. In groups A and B, the patients with fractures <60 years mainly included young and middle-aged men, and the proportion of fractures was higher in men than in women. The number of lumbar fractures in women >60 years increased significantly, and the proportion of affected women was higher than that of men. Young and middle-aged people predominate the working class, where the main outdoor high-risk workers are men who perform tasks such as aerial work and transportation, and thus, have greater fracture risks. Therefore, these age groups have to be given special attention, and preventive measures need to be taken when necessary to avoid fractures. With the progress of society and the optimization of medical conditions, aging has become a major factor contributing to lumbar fractures among the elderly. The proportion of elderly women in group B (>60 years old) was significantly higher than that in group A, which was probably due to lumbar fractures in elderly women and osteoporosis caused by the decrease in estrogen level after menopause.^[10–12]^

From the results of the analysis of the distribution of injury factors, we found that traffic accidents are the main cause of injury, which is consistent with the results of previous epidemiological studies.^[13–15]^ Traffic accident injury is the main factor in traffic accidents. The lumbar spine is affected by a direct impact. For example, the impact site of a pedestrian is damaged when a car hits them. Because the impact point is below the center of gravity of the pedestrian, the pedestrian rotates centripetally and bounces to the top of the vehicle, resulting in 1 or multiple fractures in this area. Table 3 shows that although the composition of patients in group B with high fall injury decreased compared to that of patients in group A, it is still an important injury-causing factor in high-energy injuries. Some studies have shown that for a height difference of more than 3 m the spinal fracture can be of different types, depending on the height of the falling individual and the location of stress, as the stress on the spine is different at different landing positions or under the shelter of the air objects. With the rapid development of the transportation and construction industry in China, high-energy injuries, such as car accident injuries and high fall injuries, have increased the proportion of spinal fractures, which brings a heavy burden to patients, families, and society. Therefore, preventing spinal fractures caused by car accident injury and high fall injury is very important. This study showed that in the incidence of injury in female patients of group B, low-energy injuries are the main injury-causing factors, especially high falls and stumbling injury, which might be related to the increase in the risk of osteoporotic fracture with the change in the lifestyle and aging.^[16]^ Therefore, the number of hospitalized patients >60 years is increasing. Postmenopausal osteoporosis is a serious problem in elderly women. As the level of collagen decreases, bone strength and toughness decrease, and brittleness increases; brittle fractures can occur easily under nontraumatic external force or due to minor trauma.^[17–19]^ As one of the most serious and common complications of osteoporosis in social medicine, osteoporotic fracture causes heavy psychological and economic burdens to patients, families, and society.

The analysis of the quarterly distribution table showed that the high incidence period of lumbar fracture was from April to June and July to September, and the proportion of affected women was higher than that of affected men. This might be related to the increase in the number of people working in this location. British researchers found that fractures occur more commonly from May to October,^[20]^ but some researchers argue that senile fractures occur more frequently in winter, probably due to the cold.^[21]^ Therefore, hospital managers and corresponding departments should timely and reasonably coordinate human, financial, and material resources for medical treatment in hospitals according to the seasonal characteristics of the peak times of fractures.

According to the distribution characteristics of the Denis classification for the injury mechanism of the 2 groups of patients, burst fracture was the main fracture type for high-energy injuries. This type of fracture occurs when the central column of the spine suffers an injury, and the posterior vertebral fracture blocks, together with the intervertebral disc tissue, which often penetrates the spinal canal and occupies it.^[22]^ Among them, spinal burst fracture is a serious lumbar vertebral fracture. A thoracolumbar vertebral fracture destroys the stability of the spine. As the key part of nerve function injury, the insertion of bone fragments into the spinal canal may further aggravate spinal cord injury, greatly impairing the normal life of patients.^[23]^ This study showed that compression fractures have become the dominant fracture type in recent years, and fall injuries and compression fractures caused by falls have become the prominent fracture type, followed by automobile accident injuries. With the rapid change in the human lifestyle and aging, lumbar fracture caused by osteoporosis has been given much attention by surgeons. Due to osteoporosis and aging, the surface density of bone trabecula decreases, and the morphological structure is also affected.^[24]^ Therefore, after experiencing slight external forces such as falls, sprains, and even mild actions such as sneezing and water falling on the body, the stress is transmitted to the spine, resulting in fracture of a vertebral bone trabecula in the spine and vertebral fracture. Our results showed that in the past 5 years, the proportion of female patients with fall injury was higher than that of male patients, and the average age was >50 years. This suggested that the proportion of female patients was more than that of male patients, and the average age was higher. Considering that such an injury is relatively rare, middle-aged and elderly female patients mostly have osteoporosis, and thus, even minor injuries cause lumbar fractures. Therefore, middle-aged and elderly patients, especially female patients, should pay attention to the prevention and treatment of osteoporosis.^[25]^

To summarize, this study determined the relationship among gender, age, fracture mechanism, and fracture time related to adult lumbar fracture in patients admitted to our hospital during the past 10 years. Lumbar fractures occur mainly in young and middle-aged people <60 years, especially males. Comparison of data after 5 years showed that the number of fractures increased, and the number of women with lumbar fractures showed an increasing trend. Lumbar fracture caused by a car accident was the main factor that affected the lumbar vertebrae. The results of this study showed that a high incidence of fractures in patients occurred from April to June and from July to September, and the proportion of injured females was higher than that of injured males. For elderly female patients, the fracture rate is gradually increasing, and impacts of different energy levels can cause fractures.^[26]^ The increasing fracture rates in older women might be related to osteoporosis caused by the decrease in the estrogen level after menopause.

This study determined the characteristics and epidemiological trends of adult lumbar fractures and provided some reference for the prevention and treatment of fractures at this location. This study has some limitations: the fracture segments, complications, and treatment plan of lumbar fractures were not determined; the length of hospital stay, cost, and outcome of patients were not investigated, which prevented us from objectively analyzing the comprehensive situation of lumbar fractures. Additionally, the number of cases in this study was relatively small, and it was a single-center study, which did not represent all the patients with a lumbar fracture in this region. To increase the accuracy of future studies, more centers and participants should be included.

## Author contributions

**Data curation:** Jianqing Ma, Xin Qiao.

**Formal analysis:** Xiufang Mo.

**Investigation:** Dongwei Wu, Ke Yang.

**Methodology:** Zhanfeng Song.

**Writing – original draft:** Dingding Jia.

**Writing – review & editing:** Zhanyong Wu.
